# Editorial: Training, performance and rehabilitation in water-based sports

**DOI:** 10.3389/fphys.2023.1279596

**Published:** 2023-09-01

**Authors:** Fabrice Joulia, Daniel Daly, Alexandre Guimard

**Affiliations:** ^1^ Center for Cardiovascular and Nutrition Research C2VN, INSERM 1263 INRAE 1260 Aix Marseille Université, Marseille, France; ^2^ UFR STAPS, Toulon, France; ^3^ Department of Movement Sciences, Faculty of Movement and Rehabilitation Sciences, KU Leuven, Leuven, Belgium; ^4^ Université Sorbonne Paris Nord, Hypoxie et Poumon, H&P, INSERM, UMR 1272, Bobigny, France; ^5^ Département STAPS, Université Sorbonne Paris Nord, Bobigny, France

**Keywords:** immersion, aquatic environment, swimming, recovery, rehabilitation, performance, sex, cold-water

Various physical and sporting activities involve immersion in water, and therefore confrontation with the specific constraints of the aquatic environment. Resistance to movement and forward motion is thus increased, and competitive and recreational swimming is the prime example. For this reason, and because it has an impact on the metabolism and general physiology of living organisms, water immersion is a physical activity frequently used to achieve specific goals, including in the field of performance. Moreover, by counteracting the effects of gravity (Archimedes’ buoyancy principle), immersion allows for the execution of muscular activity with reduced joint constraints, making it beneficial for rehabilitation purposes. The aquatic environment is also frequently used for recovery, as in the case of cold-water immersion to promote thermal losses. Finally, immersion appears to have a relaxing effect, justifying its application for therapeutic purposes. The Research Topic focuses on aspects of physiology, training, performance enhancement and rehabilitation in and through physical and sporting activities in water.

## The Research Topic articles

The aquatic environment is conducive to various forms of physical activity. Due to the increased resistance to movements, its counterbalancing effect on gravity, and its thermal conductivity, water immersion is also regularly used in the context of rehabilitation or recovery after sport and exercise. It has been shown that sex has a significant impact on sports performance. During swimming an increase in energy cost with distance was higher for men than women independent of the differences in body composition (Massini et al., 2021). In their review comparing several sports, Bassett et al. (2020) found an advantage of around 9% for males during swimming without distinction for distance and swimming stroke. In their study of swimming starts, Rudnik et al. found no significant differences between men and women’s reaction time but the total start time (15-m), the duration of component parts (5 and 10-m), and almost all of the time periods of starting phases were shorter in male swimmers. This study defines which variables should be considered while evaluating start performance in male and female swimmers separately. The management of swimming training planning must not only take sex into account, but also age. As participation in swimming can start at a very young age and continue into the advanced years, because it is an “unloaded” sport (i.e., in relative weightlessness), the question of early specialization arises. By comparing the model of performance progression (between 2006 and 2017) for countries grouped by continent, Yustres Amores et al. showed that, with the exception of America, swimmers who have already taken part in the world junior championships perform better in the same competition in the senior category than their counterparts who have not.

Because of the increase in competitiveness in high-level sport, any increase in performance, however small can make the difference during high level competition. Since it contains high levels of inorganic nitrate (NO_3_^−^) beetroot juice increases blood nitric oxide concentration promoting capillary vasodilatation and muscle perfusion. Consequently beetroot is a dietary supplement regularly used by participants in various physical activities (Domínguez et al., 2018). However, in swimming, there is no clear data on the effects of beetroot juice on performance. Surprisingly, Moreno et al. found no change in physiological parameters recorded during a 6 m × 100 m swim series after beetroot ingestion. Nevertheless, despite no physiological changes they observed an increase in the performance of the final repetition suggesting an effect on fatigue after beetroot ingestion. To enhance recovery, land-based sports such as soccer make frequent use of immersion, particularly in cold water. It has been argued, for example, that this mode of recovery can limit the inflammatory effect of exercise, however its effectiveness remains ambiguous (Ihsan et al., 2016) and placebo effects to be considered. Following a soccer match simulation (Loughborough Intermittent Shuttle Test), the results of Nasser et al. suggest improved recovery kinetics for muscle damage markers and physical performance in cold water immersion and placebo compared to resting conditions. The placebo effect could therefore at least partially explain the effectiveness of cold water immersion (Nasser et al.).

Exercise in an aquatic environment is regularly used during rehabilitation. To increase resistance, specific equipment can be employed. Increasing such resistance improves balance and walking ability, which are associated with the prevention of falls (Katsura et al., 2010). In their meta-analysis Dai et al. screened nearly 700 articles. Based on 10 RCTs which met their inclusion criteria, they concluded that exercise in such an environment improved balance, walking ability, and quality of life in patients with Parkinson’s disease as compared to land based training but there was no difference in motor functions reflected by lower limb strength. It is difficult to quantify the load applied to the foot when performing movements in water. Using different types of leg resistance fins Gislason et al. showed that progression of additional external forces can be estimated using surface area and/or angular velocity.

Training, performance and rehabilitation in aquatic sports requires land based beings (athletes and patients) to adapt to the specific properties of the aquatic environment. Integrative research is needed to deepen our knowledge in this field and continue to offer practical applications.
